# Gut jet lag: how circadian rhythm disruption undermines the Chrono-Microbiota-Motility axis and induces functional constipation

**DOI:** 10.3389/fnut.2025.1678482

**Published:** 2025-10-02

**Authors:** Jitong Li, Keying Yu, Xiaodan Sui, Houbo Deng, Yan Leng, Tiejun Liu

**Affiliations:** ^1^Changchun University of Chinese Medicine, Changchun, China; ^2^Affiliated Hospital of Changchun University of Chinese Medicine, Changchun, China

**Keywords:** functional constipation, circadian rhythm, gut microbiota, gut jet lag, chronotherapy

## Abstract

Functional constipation (FC) is a highly prevalent gastrointestinal disorder globally. Its complex and multidimensional pathophysiological mechanisms and the limitations of conventional treatments highlight the urgent need to explore new theoretical frameworks. Chronobiological research reveals that the circadian rhythm system plays a central role in maintaining physiological homeostasis, and rhythm disruption caused by modern lifestyles is becoming a common driving factor for various chronic diseases. The gut microbiota, as a key regulator of host physiology, its intrinsic rhythmicity and synchronization with the host’s biological clock are crucial for gut health. This review systematically integrates cross-disciplinary evidence from chronobiology, microbiology, and gastrointestinal motility. Based on this evidence, we propose and substantiate the Chrono-Microbiota-Motility axis as a novel theoretical framework. This framework unveils a novel pathogenic mechanism: gut jet lag. This term describes a state of desynchronization between host and gut microbiota rhythms. This desynchronization is caused by modern lifestyle factors such as irregular routines, disordered eating times, and nocturnal light exposure. This desynchronization translates into gut dysfunction through three core pathways: disrupted short-chain fatty acid production rhythm weakens intestinal propulsive motility, dysregulation of the intrinsic gut clock inhibits morning high-amplitude contractions, and impaired intestinal barrier function triggers low-grade inflammation and increased sensitivity. Based on this theoretical framework, this paper systematically elucidates the scientific basis of “chronotherapy” as a non-pharmacological intervention strategy, including multi-dimensional synergistic interventions such as time-restricted eating, regular routines, light management, and individualized supplement application. This theory achieves a paradigm shift in the etiological research of FC from material homeostasis to temporal synchronization, providing a new explanatory framework for refractory cases, opening up fundamental treatment pathways, and offering evidence-based guidance for the formulation of modern lifestyle health guidelines. Incorporating the time dimension into FC research and clinical practice represents a key breakthrough in understanding this complex disease. Furthermore, it reflects modern medicine’s shift toward a systemic, individualized, and preventive approach.

## 1 Introduction

Functional Constipation (FC), as a highly prevalent gastrointestinal disorder, imposes a heavy medical and economic burden globally. Systematic reviews and meta-analyses show that the global pooled prevalence of FC is approximately 15.3%, with significant variations across different regions, ages, and genders (1). Its pathophysiological mechanisms are considered complex and multidimensional, involving genetic susceptibility, dietary structure, lifestyle, and intrinsic intestinal motility abnormalities.

However, a critical clinical observation remains: current mainstream treatments often fall short. Strategies such as increasing dietary fiber, using various laxatives, and prokinetics can partially alleviate symptoms. Despite this, they are ineffective or unsustainable for a considerable proportion of patients, leading to high recurrence rates (2). This heterogeneity and limitation in treatment response strongly suggest that existing theories may not fully cover the core etiology of FC. This implies that our understanding of the fundamental, systemic pathophysiological mechanisms driving the disorder is incomplete. Consequently, a new theoretical framework is urgently needed.

Meanwhile, modern societal lifestyles, such as shift work, irregular eating times, and excessive nocturnal light exposure, are increasingly and commonly disrupting the human circadian rhythm system. Extensive evidence indicates that such rhythm disruption is a common risk factor for various chronic diseases, including obesity, type 2 diabetes, and cardiovascular diseases (3). Chronobiology research has confirmed that a stable circadian rhythm system is crucial for maintaining core physiological processes such as metabolism, immune response, and cell repair. The gut microbiota is a key regulator of host physiology. Its intrinsic rhythmicity and its synchronization with the host’s biological clock are essential for maintaining intestinal homeostasis. The composition and function of the microbiota are profoundly influenced by various factors such as genetics, diet, and environment, and rhythm disruption is one of the key environmental factors that disrupt this homeostasis (4). Modern lifestyle habits, such as irregular eating times and nocturnal light exposure, have been shown to directly disrupt the rhythm of the gut microbiota, thereby affecting host health (5). However, systematically linking these findings to explore how rhythm disruption triggers FC by undermining the “host-microbiota” time axis remains an emerging area that warrants deeper exploration.

The purpose of this paper is to systematically integrate and analyze existing cross-disciplinary evidence to, for the first time, comprehensively construct a theoretical framework for explaining the etiology of FC—the Chrono-Microbiota-Motility axis. The theory posits a dynamic network interconnecting the host’s circadian system (Chrono), the rhythmic activity of the gut microbiota (Microbiota), and intestinal motility. It underscores that temporal synchronization among these three elements is critical for normal gut function, whereas their desynchronization constitutes the core pathophysiological mechanism of FC. The significance of this paper lies in its attempt to fill a critical gap in current FC etiological research. It provides a biologically plausible new mechanistic framework to explain cases unresponsive to conventional treatments. Moreover, it fundamentally challenges the traditional notion of FC as a static, time-independent physiological disorder.

Therefore, this paper aims to make contributions at the following three levels:

1. **Theoretical level**: To propose and substantiate gut jet lag—i.e., host-microbiota rhythm desynchronization—as an independent and important pathophysiological mechanism of FC, thereby expanding the etiological research of FC from material and structural dimensions to systemic temporal coordination dimensions.
2. **Clinical level**: Based on this theoretical framework, to propose new clinical assessment directions (e.g., collection of “chronobiological history”) and systematically elaborate non-pharmacological intervention strategies targeting the restoration of rhythm synchronization, namely “Chronotherapy.”
3. **Public health level**: To provide detailed scientific explanations for the negative impact of modern lifestyles such as irregular routines on gut health, offering evidence-based support for revising and improving relevant public health guidelines.

## 2 Composition, dysregulation, and pathogenic mechanism of the Chrono-Microbiota-Motility axis

**Figure 1:**
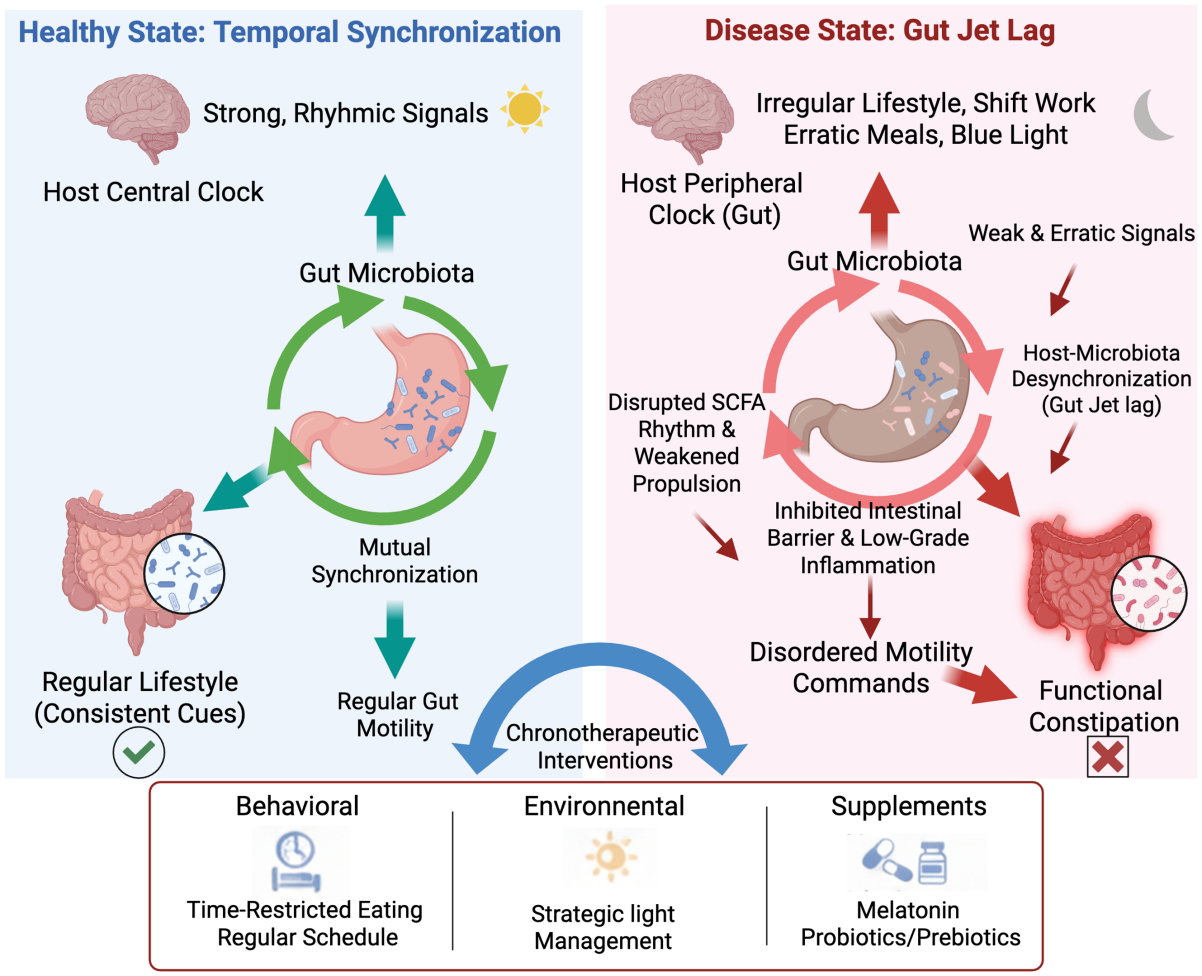
The role of host-microbiota desynchronization in the pathogenesis of functional constipation and the mechanism of chronotherapeutic interventions.

The Chrono-Microbiota-Motility axis is a complex regulatory network, the stability of which is essential for maintaining normal gut function. This section will elaborate on the composition of this axis and its two primary functional states: homeostasis, which is maintained by temporal synchronization, and dysregulation, also termed gut jet lag, which leads to FC. Figure 1 provides a schematic overview of this model, contrasting the synchronized and desynchronized pathways and introducing the chronotherapeutic strategies designed to restore balance.

The schematic diagram illustrates the contrast between a healthy, synchronized state and a diseased, desynchronized state of the gut clock. (Left Panel) In a healthy state of “Temporal Synchronization,” the host’s central clock, entrained by regular lifestyle cues, sends strong rhythmic signals that promote mutual synchronization with the gut microbiota. This coordinated rhythmicity ensures regular gut motility and maintains overall gut health. (Right Panel) In the disease state of gut jet lag, irregular lifestyles (e.g., shift work, erratic meals, nocturnal blue light exposure) disrupt the peripheral clock in the gut, leading to a desynchronization between the host and the microbiota. This results in weak and erratic signals and downstream pathological consequences, including disrupted SCFA rhythms, weakened propulsion, an inhibited intestinal barrier, and low-grade inflammation. These factors collectively contribute to disordered motility commands, ultimately culminating in functional constipation. (Bottom Panel) chronotherapeutic interventions, encompassing behavioral (e.g., time-restricted eating), environmental (e.g., strategic light management), and supplemental (e.g., melatonin, probiotics/prebiotics) approaches, are proposed to restore synchronization and alleviate symptoms. Abbreviation: SCFA, short-chain fatty acids.

### 2.1 Axis homeostasis: temporal synchronization as the basis for normal gut motility

The homeostasis of the Chrono-Microbiota-Motility axis, as depicted in Figure 1, is central to maintaining normal intestinal physiological function, especially regular motility. This homeostasis relies on the precise and dynamic coordination among its three major internal components—the host circadian rhythm system, the gut microbiota, and the intestinal motility system. The core mechanism lies in temporal synchronization. This principle dictates that the rhythmic activity of the gut microbiota must align with the host’s own biological clock. Together, they jointly drive and regulate the periodic changes in intestinal motility. This synchronization ensures that key physiological processes, such as digestion, absorption, and defecation, occur efficiently within optimal time windows. The intestinal motility system is a complex network comprising the enteric nervous system (ENS), interstitial cells of Cajal (ICCs), and smooth muscle cells. It does not merely execute commands passively. Instead, this system exhibits its own intrinsic rhythmicity and actively integrates temporal signals from both the host and the microbiota. It thus serves as the crucial effector that ensures the coordinated function of the entire axis.

There is a complex and precise bidirectional regulatory relationship between the host circadian rhythm system and the gut microbiota. The host’s circadian system includes a central clock in the suprachiasmatic nucleus and peripheral clocks in organs like the intestine. These clocks collectively create a rhythmic environment for the gut microbiota. They achieve this by regulating key physiological processes, including body temperature, hormone secretion (e.g., cortisol and melatonin), immune responses, and intestinal epithelial cell renewal (6, 7). This rhythmic environment selects and shapes the composition and function of the gut microbiota, leading to significant 24-h periodic fluctuations in the abundance of many key microbial groups and their metabolites [e.g., short-chain fatty acids (SCFAs), secondary bile acids] (8). The rhythmic activities of these microbial communities form the basis for the normal function of the Chrono-Microbiota-Motility axis.

On the other hand, the gut microbiota and its metabolites can also regulate the host’s biological clock in reverse, forming a feedback loop. SCFAs produced by the microbiota, especially butyrate, are not only the primary energy source for colon epithelial cells but can also directly affect the expression rhythm of core clock genes in intestinal cells through epigenetic modifications (e.g., histone acetylation) (9, 10). This “temporal signal” from microorganisms can fine-tune or even partially reset peripheral biological clocks, synchronizing them with the metabolic rhythms of the microbiota. When this synchronization is maintained, the intestinal motility system receives consistent, coordinated rhythmic commands from both host and microbiota (4). SCFAs produced by the microbiota at specific times can stimulate enterochromaffin cells (EC cells) to release 5-hydroxytryptamine (5-HT), which is a key neurotransmitter for initiating intestinal peristaltic reflexes and high-amplitude propulsive contractions (HAPCs) (11, 12). Therefore, a microbiota that is temporally synchronized with the host is a direct executor and amplifier for ensuring rhythmic intestinal motility and regular defecation.

### 2.2 Axis dysregulation: gut jet lag as a core pathophysiological mechanism of FC

Gut jet lag refers to the state of desynchronization between the host’s biological clock and the gut microbiota rhythm, which is the core manifestation of the Chrono-Microbiota-Motility axis dysregulation. Modern lifestyles, such as irregular eating times (e.g., skipping breakfast, late-night eating) and excessive exposure to artificial light at night (especially blue light), are the primary external driving factors leading to gut jet lag (5, 13). These factors, directly or indirectly, disrupt the originally coordinated temporal signals between the host and microbiota, triggering a cascade of reactions that ultimately lead to FC.

Irregular eating times are the most direct factor disrupting microbiota rhythms. The composition and metabolic activity of the gut microbiota are highly dependent on the host’s nutrient intake. When eating times are inconsistent with the “feeding window” expected by the host’s biological clock, the microbiota receives chaotic temporal signals. For example, extensive eating at night, when the body should typically be in a fasting and repair state, forces bacteria active during the day and responsible for digesting carbohydrates and proteins to start working at the wrong time, while those adapted to the nocturnal environment are inhibited. This “misaligned” metabolic activity not only alters the day-night rhythm of the microbiota but also leads to dysregulation in the production rhythm of its metabolites (such as SCFA), thereby affecting intestinal motility (5, 13).

Nocturnal artificial light exposure, on the other hand, acts through a more indirect but equally powerful mechanism. Light is the strongest signal synchronizing the host’s central biological clock. Nighttime light, especially blue light from electronic screens, inhibits the pineal gland’s secretion of melatonin. Melatonin is not only a key hormone regulating sleep but also an important “temporal signal” that conveys “darkness” information to peripheral biological clocks throughout the body, including the intestine. A decrease in melatonin levels has significant consequences. It directly disrupts the biological clock of intestinal epithelial cells. Furthermore, it may indirectly alter the microbiota’s survival environment by affecting the intestinal immune system and permeability. Ultimately, these changes lead to the dysregulation of microbiota rhythms (14). Therefore, gut jet lag is not an isolated phenomenon but a natural consequence of modern lifestyles acting on the Chrono-Microbiota-Motility axis. Once this desynchronized state forms, it translates upstream rhythm disruption into downstream intestinal motility disorders through the following three main pathways, ultimately manifesting as FC.

Similar to physiological “jet lag,” we propose the concept of gut jet lag to describe intestinal motility disorders caused by desynchronization between the host’s biological clock and gut microbiota rhythms. This constitutes the core pathophysiological mechanism of FC at the chronobiological level. In modern society, various common lifestyle factors, such as irregular routines (shift work, staying up late), transmeridian travel, irregular eating times, and high-fat/high-sugar diets, continuously disrupt the host’s endogenous biological rhythms. These factors pose a continuous challenge to the Chrono-Microbiota-Motility axis and are major drivers of gut jet lag Existing research clearly indicates that shift work is closely associated with a higher incidence of functional bowel symptoms (including constipation), and its underlying mechanism points to circadian rhythm disruption altering the gut microbiome (15).

Irregular eating times and nocturnal eating behaviors are more common and insidious rhythm disruptors. Eating is the strongest signal for synchronizing the gut’s biological clock, and the regularity of its timing is crucial. When eating times become random or conflict with the host’s endogenous rhythms (such as the sleep–wake cycle), the intestine and microbiota lose reliable external time references and cannot anticipate when the next wave of nutrients will arrive. This “temporal uncertainty” weakens the effectiveness of “eating” as a synchronizing signal, leading to disordered or flattened rhythmic oscillations of the gut microbiota and potentially impairing the integrity of the intestinal barrier (5, 13). Studies have confirmed that irregular eating patterns are closely related to gut dysbiosis, disruption of SCFA synthesis rhythms, and metabolic disorders. Conversely, synchronizing eating times with endogenous circadian rhythms, such as adopting early time-restricted feeding (eTRF), can enhance microbiota diversity and function and improve metabolic health (16). Therefore, the widespread irregular eating habits in modern life, by disrupting the rhythmicity of the microbiota, directly lead to functional disorders of the Chrono-Microbiota-Motility axis, which is key for the gut jet lag theory to explain a broader FC population.

Furthermore, nocturnal artificial light exposure (ALAN) interferes with the entire system from upstream. ALAN directly weakens the signal strength of the central clock by inhibiting the secretion of melatonin, a key “darkness signal,” thereby indirectly affecting its ability to regulate peripheral intestinal clocks (17). This internal biological clock desynchronization caused by light pollution is considered one of the core mechanisms leading to a high incidence of metabolic diseases in groups such as shift workers. Carefully regulating light exposure (e.g., avoiding blue light exposure at night) to adjust melatonin peaks is a potential intervention strategy for recalibrating the biological clock, improving sleep, and metabolic health (18).

In summary, gut jet lag is a complex pathological state resulting from the synergistic action of various modern lifestyle factors, including shift work, irregular eating, and nocturnal light exposure. These factors collectively cause the host and microbiota to transition from “synchronized dancers” to a “chaotic combination” with inconsistent steps, thereby systematically inducing intestinal motility disorders.

### 2.3 Pathogenic mechanism of gut jet lag: how desynchronization translates into constipation

Gut jet lag translates upstream rhythm desynchronization into downstream intestinal motility disorders through three interconnected core mechanisms, ultimately leading to the occurrence of FC.

#### 2.3.1 Disrupted SCFA production rhythm and weakened intestinal motility

SCFAs are key metabolites produced by gut microbiota fermenting dietary fiber. They not only provide energy for intestinal epithelial cells but are also important signaling molecules regulating intestinal motility. Under physiological conditions, SCFA production and concentration exhibit clear circadian rhythms. This rhythmic signal can precisely act on colonic smooth muscle cells and the enteric nervous system, driving the colon to produce rhythmic, efficient propulsive contractions. However, gut jet lag disrupts the microbiota’s rhythm, directly leading to the loss of the inherent rhythmicity in SCFA production. Studies show that irregular eating patterns significantly alter the composition and function of the gut microbiome, thereby disrupting the synthesis rhythm of SCFAs (especially butyrate and propionate) (14). When SCFA signals become chaotic or remain at low levels, their driving effect on intestinal motility weakens, leading to slow colonic transit, fecal retention, and ultimately constipation.

#### 2.3.2 Intestinal biological clock dysregulation and decreased propulsive motility

The intestine itself possesses an independent biological clock system that regulates the rhythm of intestinal movements, especially the efficient HAPCs in the morning, which are crucial for defecation. The intestinal biological clock stays synchronized with the central biological clock to ensure that intestinal motility is strongest during the active phase (daytime). Factors such as nocturnal artificial light exposure and irregular eating times directly interfere with the normal rhythm of the intestinal biological clock. Nocturnal light inhibits melatonin secretion, weakening the “darkness” signal transmitted to the intestine, while nocturnal eating gives the intestine a false “feeding” signal, activating it when it should be in a resting state (19). This chaotic temporal signal leads to the loss of the intrinsic motility rhythm of the intestine, particularly weakening morning propulsive motility, causing patients to feel “no urge to defecate in the morning” and experience difficulty in defecation, which is a typical complaint of FC patients.

#### 2.3.3 Impaired intestinal barrier function and low-grade inflammation

The integrity of the intestinal barrier is also precisely regulated by circadian rhythms. In a state of gut jet lag, desynchronization between host and microbiota rhythms can impair intestinal barrier function. Studies clearly indicate that disordered eating times can disrupt the expression of tight junction proteins in intestinal epithelial cells, leading to increased intestinal permeability, i.e., “leaky gut” (9). After barrier damage, bacterial lipopolysaccharides (LPS) and other endotoxins in the intestinal lumen are more likely to enter the bloodstream, triggering a low-grade systemic inflammatory response. This chronic inflammation, driven by rhythm disruption, can further impair intestinal neuromuscular function and may lead to visceral hypersensitivity, exacerbating symptoms such as bloating and abdominal pain, forming a vicious cycle of “motility disorder-barrier damage-inflammation-motility worsening,” making constipation more complex and persistent.

## 3 Clinical assessment and intervention for gut jet lag

**Figure 2:**
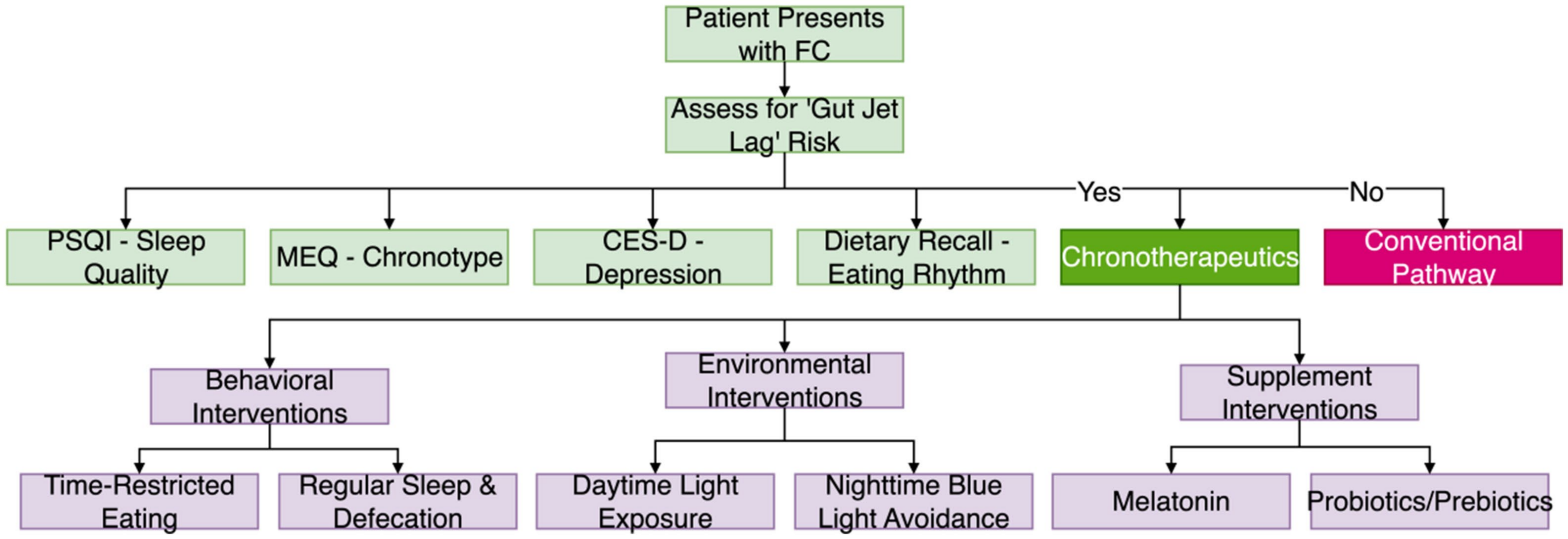
Clinical workflow for gut jet lag assessment and intervention. This flowchart outlines the proposed clinical pathway, from initial patient assessment to the branching decision between chronotherapeutic and conventional interventions. PSQI, Pittsburgh Sleep Quality Index; MEQ, Morningness-Eveningness Questionnaire; CES-D, Center for Epidemiologic Studies Depression Scale.

Identifying and quantifying gut jet lag is a prerequisite for precise intervention. Due to its multifactorial nature, assessment should combine subjective questionnaires and objective biomarkers to comprehensively capture the patient’s lifestyle, rhythm status, and intestinal function. The proposed clinical pathway, from initial screening to the implementation of chronotherapy, is illustrated in Figure 2.

### 3.1 Subjective assessment: combined application of multi-dimensional questionnaires

Identifying and quantifying gut jet lag is a prerequisite for precise intervention. Its assessment should combine subjective questionnaires and objective biomarkers to comprehensively capture the patient’s lifestyle, rhythm status, and intestinal function. Subjective questionnaires, as a rapid and non-invasive cornerstone for screening gut jet lag risk, are clinically recommended to be used in combination with various validated standardized questionnaires for multi-dimensional assessment. In terms of sleep and rhythm assessment, the Pittsburgh Sleep Quality Index (PSQI) can comprehensively evaluate the patient’s sleep quality, sleep onset latency, sleep duration, sleep efficiency, and daytime dysfunction over the past month. A total score > 5 usually indicates poor sleep quality, which is an important signal of gut jet lag (20); while the Morningness-Eveningness Questionnaire (MEQ) is used to assess an individual’s “chronotype,” i.e., their intrinsic biological rhythm preference. Studies show that evening types are more prone to irregular eating and sleep deprivation, thereby increasing the risk of gut jet lag (21). In terms of emotional state assessment, given the close bidirectional relationship between emotional disorders, gut microbiota, and circadian rhythms, using the Center for Epidemiologic Studies Depression Scale (CES-D) to screen for depressive symptoms helps identify psychological factors that may exacerbate gut jet lag (22). Dietary behavior assessment is also crucial. Tools like food frequency questionnaires (FFQ) or 24-h dietary recalls, combined with eating time records, can assess the patient’s dietary structure. Importantly, these methods can also determine if the eating rhythm is disrupted by behaviors such as skipping breakfast or late-night eating. This is critical because irregular eating patterns are a core driver of gut microbiota rhythm dysregulation (23). Through the combination of these questionnaires, clinicians can systematically assess the presence of gut jet lag and its potential inducing factors from multiple dimensions such as sleep, emotion, diet, and individual rhythm preferences, providing a solid basis for subsequent individualized interventions.

### 3.2 Objective assessment: searching for biomarkers of rhythm desynchronization

To complement subjective findings and achieve a more accurate diagnosis of gut jet lag, objective biomarkers are essential. These markers span multiple biological levels, reflecting the complex nature of the Chrono-Microbiota-Motility axis. They can be broadly grouped into several key categories: (1) markers of the central clock, such as core rhythmic hormones; (2) indicators of the peripheral microbiota clock, including microbial metabolites; (3) physiological markers reflecting systemic rhythmicity, like autonomic nervous system activity; and (4) molecular markers related to gene expression, intestinal barrier integrity, and low-grade inflammation. Supplementary material S1 provides a comprehensive, detailed summary of these potential biomarkers.

### 3.3 Intervention strategies: multi-dimensional resetting of the “biological clock”

Based on the gut jet lag theory, the core of intervention strategies for FC is to resynchronize the biological rhythms of the host and gut microbiota through multi-dimensional, synergistic approaches, restoring the homeostasis of the Chrono-Microbiota-Motility axis. These interventions, namely “chronotherapy,” primarily use non-pharmacological means to adjust lifestyle and environmental factors to reset the disordered biological clock. The main strategies include behavioral interventions, environmental interventions, and supplement interventions.

#### 3.3.1 Behavioral interventions: rebuilding regular “time signals”

Behavioral intervention is the cornerstone of “chronotherapy,” with its core being to recalibrate the intrinsic biological clock by establishing stable, predictable external time signals. Among these, time-restricted eating (TRE) and regular routines are two key methods.

**TRE** provides clear, strong “feeding/fasting” rhythm signals for the gut microbiota by limiting daily eating to a fixed window. During fasting, the intestine can initiate migrating motor complex (MMC) to effectively clear intestinal contents, preparing for the next meal. Concentrating most daily caloric intake during the active phase of the day (daytime) and ensuring a sufficiently long fasting period at night has been proven to significantly improve the composition and rhythmicity of the gut microbiota, enhance its diversity, and optimize the production rhythm of SCFAs (24). By aligning the eating window with the host’s active phase (daytime), TRE can effectively correct gut jet lag caused by irregular eating, thereby enhancing colonic propulsive motility and improving constipation symptoms.

**Regular routines and bowel training** aim to strengthen the stability of the sleep–wake cycle. Going to bed and waking up at fixed times daily helps stabilize the secretion rhythms of melatonin and cortisol, thereby strengthening the central biological clock’s ability to synchronize peripheral intestinal clocks. In addition, combining morning bowel training (i.e., attempting defecation at a fixed time after breakfast) can utilize the gastrocolic reflex and the physiological peak of morning HAPCs to re-establish regular bowel habits. The establishment of this behavioral pattern is an important part of resynchronizing the Chrono-Microbiota-Motility axis.

#### 3.3.2 Environmental interventions: harnessing the power of light

Light is the strongest external signal synchronizing the central biological clock. By strategically managing the light environment, biological rhythms can be effectively regulated.

**Enhanced daytime light exposure**, especially receiving sufficient natural light or high-intensity blue light in the morning, can effectively inhibit melatonin secretion, increase alertness, and strongly synchronize the central biological clock. This clear “daytime signal” helps establish a stable circadian rhythm, thereby better regulating downstream intestinal biological clocks.

**Avoiding blue light at night** is equally important. Excessive exposure to artificial light at night, especially blue light from electronic screens such as mobile phones and computers, severely inhibits the normal secretion of melatonin, leading to difficulty falling asleep and circadian rhythm disruption (25). Therefore, wearing blue-light blocking glasses, using night mode, or completely avoiding electronic devices 1–2 h before bedtime are crucial measures to protect melatonin secretion and maintain biological clock homeostasis. By carefully regulating light, sleep quality can be significantly improved, and intestinal rhythm recovery can be indirectly promoted (26).

#### 3.3.3 Supplement interventions: targeted regulation of “Chrono-Microbiota” interaction

Building upon behavioral and environmental interventions, specific supplements can serve as important auxiliary means to more directly target the interaction between the biological clock and gut microbiota.

**Exogenous melatonin** supplementation is a direct “clock reset” strategy. Melatonin is not only a key regulator of the central biological clock, but its concentration in the gastrointestinal tract is much higher than in the blood, allowing it to directly act on the enteric nervous system and intestinal epithelial cells, regulating intestinal motility and secretory function. Sleep quality is closely related to health status, and abnormal sleep duration is associated with an increased risk of mortality (27). For FC patients with sleep disorders or melatonin secretion rhythm disruption, low-dose melatonin supplementation before bedtime can not only improve sleep quality but also alleviate constipation symptoms through its direct regulatory effect on intestinal motility. Studies show that sleep deprivation affects gene expression and protein abundance in the cerebral cortex, thereby affecting overall physiological function (28). However, its optimal dosage, administration time, and long-term efficacy and safety still require more research.

The application of **specific probiotics/prebiotics** aims to indirectly calibrate the intestinal biological clock by reshaping the composition and rhythm of the gut microbiota. Probiotic preparations containing Bifidobacterium and Lactobacillus have been shown to improve gut microbiota structure, increase the production of SCFAs such as butyrate, thereby regulating intestinal motility and sensory function (29). More importantly, some studies indicate that specific probiotic supplementation can restore disordered microbiota rhythms, resynchronizing them with the host’s circadian rhythm. In addition, prebiotics such as fructooligosaccharides (FOS) and inulin, by providing “fuel” for beneficial bacteria, selectively promote their growth, can also achieve the goal of improving microbiota structure and function. Combining probiotic/prebiotic supplementation with time-restricted eating may produce synergistic effects, more effectively restoring the homeostasis of the Chrono-Microbiota-Motility axis.

## 4 Discussion and outlook

This review consolidates interdisciplinary evidence to propose the Chrono-Microbiota-Motility axis, establishing gut jet lag—a state of host-microbiota temporal desynchronization—as a key pathophysiological mechanism in FC. The primary conceptual contribution of this framework is the reframing of FC from a disorder of material homeostasis (e.g., lack of fiber) to one of temporal synchronization. This temporal framework does not negate traditional etiologies but complements them by offering an upstream, dynamic regulatory perspective. It provides a plausible explanation for refractory FC and highlights the vulnerability of populations with disrupted routines, such as shift workers (30).

The clinical implications are immediate and practical. The proposed model advocates for integrating a “chronobiological history” (e.g., sleep patterns, meal timing) into the standard diagnostic workup for FC (31, 32). It also provides a strong scientific rationale for chronotherapy—a suite of non-pharmacological interventions like TRE (33) and strategic light management (34, 35)—as a foundational treatment strategy. However, the promise of this framework is balanced by significant limitations in its current evidence base, which warrant careful consideration.

Therefore, future research must prioritize human randomized controlled trials (RCTs) to validate the causal relationship between gut jet lag and FC and to assess the efficacy of chronotherapeutic interventions. Key objectives should include developing accessible biomarkers (e.g., rhythmic profiles of salivary cortisol or fecal SCFAs) and identifying patient subtypes most likely to benefit from this approach. Ultimately, extending this temporal perspective could prove valuable for understanding and managing a broader spectrum of functional gastrointestinal and metabolic disorders, marking a critical step toward a more systemic and preventive medicine.

## 5 Evidence statement and limitations

While the Chrono-Microbiota-Motility axis offers a novel framework, its evidentiary foundation has significant limitations. The mechanistic links are largely supported by preclinical data from rodent models, and the direct translation of these findings to humans is speculative due to species-specific differences. Furthermore, existing human evidence is predominantly observational, lacking the robust causal inference that can only be provided by randomized controlled trials (RCTs). To provide full transparency on this evidence base, Supplementary material S2 categorizes all cited studies. These limitations do not diminish the framework’s potential but rather highlight the critical need for human interventional studies to validate its clinical utility.
