# Supplementary material for: Gut jet lag: how circadian rhythm disruption undermines the Chrono-Microbiota-Motility axis and induces functional constipation
Source: Front Nutr. 2025 Oct 2;12:1678482. doi: 10.3389/fnut.2025.1678482 (PMC12528175; doi:10.3389/fnut.2025.1678482)
Supplement: Supplementary file 1 [file Table_1.DOCX]

Supplementary Material 1 Potential Biomarkers for Assessing Rhythm Desynchronization and FC These biomarkers provide objective evidence for assessing rhythm desynchronization. Future research should focus on developing more convenient and cost-effective detection methods and clarify their clinical value in the diagnosis and evaluation of treatment efficacy for FC.

| **Biomarker** | **Detection Method** | **Related Rhythm Disorder/Disease** | **Key Findings** | **Reference** |
| --- | --- | --- | --- | --- |
| Fecal Calprotectin (F-calprotectin) | ELISA | Intestinal inflammation, circadian rhythm disruption, Alzheimer's disease | Circadian variability of fecal calprotectin is related to intestinal inflammation; in Alzheimer's disease, it is a biomarker associated with gut dysbiosis and circadian rhythm disruption. | ^[1, 2]^ |
| Cortisol | Saliva/Plasma testing | Stress response, circadian rhythm disruption, FC, depression | Infantile FC or abdominal pain showed no significant association with cortisol circadian rhythm and stress response; DHEAS/cortisol ratio might be lower in constipation patients, suggesting an association with depression. | ^[3]^ |
| Heart Rate Variability (HRV) | 24-hour ECG monitoring (Holter) | Fibromyalgia, autonomic dysfunction, constipation, mixed irritable bowel syndrome, major depressive disorder | Nighttime HRV abnormalities (sympathetic dominance) in fibromyalgia patients are significantly associated with constipation symptoms; circadian rhythm changes in autonomic nervous system activity in IBS patients, HRV can serve as a biomarker for assessment; reduced circadian modulation of HRV in MDD patients. | ^[4-6]^ |
| Melatonin | Saliva/Plasma testing | Sleep-wake cycle, polycystic ovary syndrome, rhythm desynchronization | Melatonin levels are abnormal in PCOS patients, a key biomarker of rhythm desynchronization; melatonin can improve skin barrier damage caused by sleep restriction, possibly mediated by gut microbiota. | ^[7, 8]^ ^[9]^ |
| Clock gene expression | RT-qPCR (leukocytes) | Obstructive sleep apnea, circadian rhythm disruption, non-alcoholic fatty liver disease | Altered expression patterns of core clock genes in OSA patients are related to disease severity; allicin improves NAFLD and gut dysbiosis by regulating clock genes. | ^[10, 11]^ |
| Plasma proteomics biomarkers | High-throughput proteomics platform | Sleep disorders, inflammation, aging | In older adults, 9 plasma protein biomarkers are associated with increased secondary sleep time, suggesting rhythm disruption or potential health issues, and inflammatory processes play an important role in regulating sleep behavior. | ^[12]^ |
| MicroRNA (miRNAs) | Plasma testing | Neurodegenerative diseases, sleep deprivation | miR-127-3p and miR-142-3p levels are significantly elevated in plasma after acute sleep deprivation, potentially linking sleep deprivation with neurodegenerative diseases. | ^[13]^ |
| Acylcarnitine | Metabolomics/Lipidomics | Chronic rhythm disruption, metabolic disorders | Propionylcarnitine levels are elevated in the liver of female rats with chronic rhythm disruption, serving as a biomarker for rhythm disruption. | ^[14]^ |
| Skin temperature rhythm | Wrist skin temperature monitoring | Mood disorders, circadian rhythm disruption | 24-hour skin temperature rhythm changes in adolescents with mood disorders. | ^[15]^ |
| SCFAs | Fecal/Blood sample analysis (GC-MS, LC-MS), SCFAs receptor expression | Intestinal motility, gut dysbiosis, depression, insulin resistance, inflammation, blood pressure rhythm | SCFA production rhythm disruption is associated with weakened intestinal motility; prebiotic chronotherapy alleviates depression by enhancing SCFA receptors and the intestinal barrier; dietary oat β-glucan improves insulin resistance by regulating colonic circadian rhythms and gut microbiome, SCFA mediates GPCRs activation; chronic stress affects SCFA circadian rhythmicity; SCFAs and their receptors play roles in sleep disorders, intestinal barrier, and blood pressure rhythm. | ^[16-21]^ |
| HD5 | Fecal testing | Sleep deprivation, gut microbiota dysbiosis | Short sleep duration is associated with decreased fecal HD5 concentration, disordered gut microbiota composition, and reduced SCFA production. | ^[20]^ |
| Intestinal barrier function biomarkers | Tight junction protein gene expression (RT-qPCR), LPS levels | Sleep deprivation, gut dysbiosis, inflammation | Acute sleep deprivation exacerbates inflammation and mental illness through gut dysbiosis and circadian rhythm disruption, and is associated with intestinal barrier dysfunction; prebiotic chronotherapy alleviates depression by enhancing SCFA receptors and the intestinal barrier. | ^[17, 22]^ |
| SERPINE1 protein | Bioinformatics analysis, in vivo and in vitro experiments | Traumatic brain injury-related depression, circadian rhythm disruption | SERPINE1 overexpression is associated with increased risk of TBI-mediated depression, possibly by disrupting circadian rhythms, and can serve as a predictive and diagnostic biomarker. | ^[23]^ |
| Liver Circadian Genes (LCGs) | Multi-omics analysis, machine learning | Liver cancer, circadian rhythm dysregulation | Liver circadian genes are widely dysregulated in liver cancer and can serve as prognostic biomarkers and therapeutic targets, e.g., RBM17. | ^[24]^ |
| Tmem87b/Fkbp1a/Ppard/Slc23a2 | Bioinformatics analysis | Hepatotoxicity, circadian rhythm disruption | These genes are identified as dual biomarkers for early detection of bisphenol B-induced hepatotoxicity, which is associated with circadian rhythm disruption and oxidative stress. | ^[25, 26]^ |
| NO, MDA, PCO, PMRS | Biochemical indicators | Aging, circadian rhythm disruption | In juvenile rats exposed to artificial light at night, these aging biomarkers are significantly elevated, suggesting that rhythm changes may accelerate the aging process. | ^[27]^ |
| DHEAS/Cortisol Ratio | Saliva/Serum testing | Depression, constipation | DHEAS/cortisol ratio may identify depression in constipation patients. | ^[28]^ |
| Trimethylamine-N-oxide (TMAO) | Detection method to be determined | Alzheimer's disease, gut dysbiosis, circadian rhythm disruption | Metabolites associated with gut dysbiosis and circadian rhythm disruption play key roles in AD pathogenesis. | ^[29]^ |
| Kynurenine | Detection method to be determined | Alzheimer's disease, gut dysbiosis, circadian rhythm disruption | Metabolites associated with gut dysbiosis and circadian rhythm disruption play key roles in AD pathogenesis. | ^[30]^ |
| Isoamylamine | Detection method to be determined | Alzheimer's disease, gut dysbiosis, circadian rhythm disruption | Metabolites associated with gut dysbiosis and circadian rhythm disruption play key roles in AD pathogenesis. | ^[31]^ |
| Gut Microbiome Composition and Metabolism | 16S rRNA sequencing, metagenomic sequencing, untargeted metabolomics | Circadian rhythm disruption, irritable bowel syndrome, chronic stress, obesity | Circadian rhythm disruption leads to reduced gut microbial diversity, altered structure and metabolic profiles, and is associated with IBS-like features and depression; the circadian rhythmicity of gut microbiome composition and metabolism is affected by chronic stress; fasting can significantly affect gut microbial diversity, composition, and metabolism. | ^[19, 32-34]^ |
| 5-HT (5-hydroxytryptamine) | Detection method to be determined | Sleep disorders, gut dysbiosis, amino acid metabolism | Gut microbiota and amino acid metabolism play key roles in 5-HT homeostasis, affecting sleep disorders. | ^[35]^ |
| Bile acid metabolites | Biochemical analysis, targeted metabolomics | Photoperiod regulation, liver metabolism | Photoperiod regulates bile acid metabolism through gut microbiota remodeling and liver gene reprogramming, and levels of certain bile acids (e.g., TCA, Tα-MCA, Tω-MCA, TCDCA, CDCA, TLCA-3S) are affected by photoperiod. | ^[36]^ |
| Host Metabolite Circulation | High-resolution metabolomics | Gut microbiome, diet composition, circadian rhythm | The gut microbiome interacts with circadian rhythm and diet composition to regulate host metabolite circulation. | ^[37]^ |
| Telomere Damage | Detection method to be determined | Aging, circadian rhythm disruption, gut dysbiosis | Non-synchronized circadian rhythm, gut dysbiosis, and telomere damage constitute the "anti-longevity triad," jointly promoting aging-related pathologies. | ^[38]^ |
| HPA axis rhythmicity | Detection method to be determined | Psychological stress, circadian rhythm disruption | The gut microbiome regulates stress response by regulating HPA axis rhythmicity. Microbiota depletion can lead to dysregulation in brain transcriptome and metabolome in stress response pathways, accompanied by glucocorticoid rhythm dysregulation. | ^[39]^ |

**Reference**

[1] Amara J, Saliba Y, Hajal J, et al. Circadian Rhythm Disruption Aggravates DSS-Induced Colitis in Mice with Fecal Calprotectin as a Marker of Colitis Severity[J]. Digestive Diseases and Sciences, 2019, 64(11): 3122-3133.

[2] Leblhuber F, Geisler S, Steiner K, et al. Elevated fecal calprotectin in patients with Alzheimer’s dementia indicates leaky gut[J]. Journal of Neural Transmission (Vienna, Austria: 1996), 2015, 122(9): 1319-1322.

[3] Kiefte-de Jong J C, Saridjan N S, Escher J C, et al. Cortisol diurnal rhythm and stress reactivity in constipation and abdominal pain: the Generation R Study[J]. Journal of Pediatric Gastroenterology and Nutrition, 2011, 53(4): 394-400.

[4] Xia Y, Zhang H, Wang Z, et al. Circadian rhythm modulation in heart rate variability as potential biomarkers for major depressive disorder: A machine learning approach[J]. Journal of Psychiatric Research, 2025, 184: 340-349.

[5] Lerma C, Martinez A, Ruiz N, et al. Nocturnal heart rate variability parameters as potential fibromyalgia biomarker: correlation with symptoms severity[J]. Arthritis Research & Therapy, 2011, 13(6): R185.

[6] Dobrek Ł, Friediger J, Furgała A, 等. [Autonomic nervous system activity in IBS patients estimated by heart rate variability (HRV)][J]. Przeglad Lekarski, 2006, 63(9): 743-747.

[7] Pundir M, Lobanova L, Papagerakis P, et al. Competitive enzyme linked aptamer based assay for salivary melatonin detection[J]. Scientific Reports, 2025, 15(1): 14276.

[8] Li W, Wang Z, Cao J, et al. Melatonin improves skin barrier damage caused by sleep restriction through gut microbiota[J]. Journal of Pineal Research, 2023, 75(1): e12874.

[9] Wang F, Xie N, Wu Y, et al. Association between circadian rhythm disruption and polycystic ovary syndrome[J]. Fertility and Sterility, 2021, 115(3): 771-781.

[10] Deng Y, Zhang Y, Xiao J, et al. Allicin Improves Diet-Induced Nonalcoholic Steatohepatitis and Gut Microbiota Dysbiosis in Mice via the Involvement of the Circadian Clock Gene Rev-erbα[J]. Journal of Agricultural and Food Chemistry, 2025, 73(15): 9019-9032.

[11] Wang H M, Shieh K R, Chang E T. Correlation of the expression of circadian-clock genes with the severity of obstructive sleep apnea in patients[J]. Chronobiology International, 2025, 42(3): 428-439.

[12] Madhawa K, Svensson T, Nt H, et al. Associations between plasma proteomic signatures and secondary sleep in older adults[J]. GeroScience, 2025, 47(3): 4623-4634.

[13] Zhang L, Grip A, Hjelmqvist D, et al. Acute Sleep Loss Increases Circulating Morning Levels of Two MicroRNAs Implicated in Neurodegenerative Disease in Healthy Young Men[J]. Journal of Cellular and Molecular Medicine, 2025, 29(7): e70523.

[14] Arora S, Houdek P, Čajka T, et al. Chronodisruption that dampens output of the central clock abolishes rhythms in metabolome profiles and elevates acylcarnitine levels in the liver of female rats[J]. Acta Physiologica (Oxford, England), 2025, 241(2): e14278.

[15] Shin M, Carpenter J S, Park S H, et al. Twenty-four-hour Skin Temperature Rhythms in Young People With Emerging Mood Disorders: Relationships With Illness Subtypes and Clinical Stage[J]. Journal of Biological Rhythms, 2025, 40(3): 262-274.

[16] Segers A, Desmet L, Thijs T, et al. The circadian clock regulates the diurnal levels of microbial short-chain fatty acids and their rhythmic effects on colon contractility in mice[J]. Acta Physiologica (Oxford, England), 2019, 225(3): e13193.

[17] Li Y, Zhang S, Li C, et al. Prebiotics chronotherapy alleviates depression-like behaviors in FMT mice through enhancing short-chain fatty acids receptors and intestinal barrier[J]. Journal of Affective Disorders, 2025: 119885.

[18] Wang X, Ye G, Wang Z, et al. Dietary Oat β-Glucan Alleviates High-Fat Induced Insulin Resistance through Regulating Circadian Clock and Gut Microbiome[J]. Molecular Nutrition & Food Research, 2024, 68(11): e2300917.

[19] Wang Y, Cui P, Cao M, et al. Chronic restraint stress affects the diurnal rhythms of gut microbial composition and metabolism in a mouse model of depression[J]. BMC microbiology, 2025, 25(1): 38.

[20] Shimizu Y, Yamamura R, Yokoi Y, et al. Shorter sleep time relates to lower human defensin 5 secretion and compositional disturbance of the intestinal microbiota accompanied by decreased short-chain fatty acid production[J]. Gut Microbes, 2023, 15(1): 2190306.

[21] Effects of prebiotics on intestinal physiology, neuropsychological function, and exercise capacity of mice with sleep deprivation - PubMed[EB/OL]. [2025-07-31]. https://pubmed.ncbi.nlm.nih.gov/36869551/.

[22] Yang D F, Huang W C, Wu C W, et al. Acute sleep deprivation exacerbates systemic inflammation and psychiatry disorders through gut microbiota dysbiosis and disruption of circadian rhythms[J]. Microbiological Research, 2023, 268: 127292.

[23] Cai Y, Huang G, Ren M, et al. Identification of preventive biomarkers associated with circadian rhythms in traumatic brain injury-mediated depression: Expression of SERPINE1 protein and bioinformatics analysis[J]. International Journal of Biological Macromolecules, 2025, 310(Pt 1): 143229.

[24] Yan J, Yang X, Lu J, et al. Development of a circadian-related prognostic signature highlights RBM17 as a stemness regulator in liver cancer[J]. Cancer Cell International, 2025, 25(1): 211.

[25] Ochiai M, Iida M, Agusa T, et al. Effects of 4-Hydroxy-2,3,3’,4’,5-Pentachlorobiphenyl (4-OH-CB107) on Liver Transcriptome in Rats: Implication in the Disruption of Circadian Rhythm and Fatty Acid Metabolism[J]. Toxicological Sciences: An Official Journal of the Society of Toxicology, 2018, 165(1): 118-130.

[26] Yue H, Hu Y, Wu X, et al. Transgenerational hepatotoxicity induced by bisphenol B as a substitute for bisphenol A[J]. Ecotoxicology and Environmental Safety, 2025, 302: 118702.

[27] Yadav G, Singh A, Trivedi A K, et al. Artificial light at night accelerates aging processes in pre-pubertal female rats[J]. Biogerontology, 2025, 26(4): 149.

[28] Sugaya N, Izawa S, Kimura K, et al. Adrenal hormone response and psychophysiological correlates under psychosocial stress in individuals with irritable bowel syndrome[J]. International Journal of Psychophysiology: Official Journal of the International Organization of Psychophysiology, 2012, 84(1): 39-44.

[29] Vogt N M, Romano K A, Darst B F, et al. The gut microbiota-derived metabolite trimethylamine N-oxide is elevated in Alzheimer’s disease[J]. Alzheimer’s Research & Therapy, 2018, 10(1): 124.

[30] Zhu H, Huang C, Luo Z, et al. Porphyromonas gingivalis Induces Disturbance of Kynurenine Metabolism Through the Gut-Brain Axis: Implications for Alzheimer’s Disease[J]. Journal of Dental Research, 2025, 104(4): 439-448.

[31] Teng Y, Mu J, Xu F, et al. Gut bacterial isoamylamine promotes age-related cognitive dysfunction by promoting microglial cell death[J]. Cell Host & Microbe, 2022, 30(7): 944-960.e8.

[32] Wu F, Guo Y, Wang Y, et al. Effects of Long-Term Fasting on Gut Microbiota, Serum Metabolome, and Their Association in Male Adults[J]. Nutrients, 2024, 17(1): 35.

[33] Hong G, Zhao Y, Zhou J, et al. Circadian rhythm perturbation causes IBS-like characteristics and altered fecal metabolome in mice[J]. BMC microbiology, 2025, 25(1): 274.

[34] Tian Y, Zhao R, Xiao S, et al. Multi-omics assessment of gut microbiota in circadian rhythm disorders: a cross-sectional clinical study[J]. Frontiers in Cellular and Infection Microbiology, 2025, 15: 1524987.

[35] Zhang C, Zhang X, Qiu H, et al. Fermented Gastrodia elata Bl. Intervenes gut microbiota and amino acid metabolism in zebrafish to promote 5-HT homeostasis against sleep disturbances[J]. Food Research International (Ottawa, Ont.), 2025, 217: 116757.

[36] Zhu H, Cao J, Ruan J, et al. Molecular mechanisms of photoperiod regulation of bile acid metabolism in Taihe silky fowls based on the gut-liver axis[J]. Poultry Science, 2025, 104(9): 105489.

[37] Zhang Y, Noya S B, Li Y, et al. The microbiome interacts with the circadian clock and dietary composition to regulate metabolite cycling in the Drosophila gut[J]. eLife, 2025, 13: RP97130.

[38] Singh S, Giron L B, Shaikh M W, et al. Distinct intestinal microbial signatures linked to accelerated systemic and intestinal biological aging[J]. Microbiome, 2024, 12(1): 31.

[39] Tofani G S S, Leigh S J, Gheorghe C E, et al. Gut microbiota regulates stress responsivity via the circadian system[J]. Cell Metabolism, 2025, 37(1): 138-153.e5.
