# Supplementary material for: Gut jet lag: how circadian rhythm disruption undermines the Chrono-Microbiota-Motility axis and induces functional constipation
Source: Front Nutr. 2025 Oct 2;12:1678482. doi: 10.3389/fnut.2025.1678482 (PMC12528175; doi:10.3389/fnut.2025.1678482)
Supplement: Supplementary file 2 [file Table_2.DOCX]

Supplementary Material 2 summary of all references

| No. | Title | Study Type | Source |
| --- | --- | --- | --- |
| 1 | Global Prevalence of Functional Constipation According to the Rome Criteria: A Systematic Review and Meta-Analysis | Systematic review and meta-analysis | Human |
| 2 | A Randomized, Multicenter, Placebo-Controlled Trial of Polyethylene Glycol Laxative for Chronic Treatment of Chronic Constipation | Randomized controlled trial | Human |
| 3 | Adverse Metabolic and Cardiovascular Consequences of Circadian Misalignment | Experimental study | Human |
| 4 | Microbiota Diurnal Rhythmicity Programs Host Transcriptome Oscillations | Experimental study | Mouse |
| 5 | Transkingdom Control of Microbiota Diurnal Oscillations Promotes Metabolic Homeostasis | Experimental study | Mouse |
| 6 | Intestinal Metagenomes and Metabolomes in Healthy Young Males: Inactivity and Hypoxia Generated Negative Physiological Symptoms Precede Microbial Dysbiosis | Experimental study | Human |
| 7 | Host Circadian Behaviors Exert Only Weak Selective Pressure on the Gut Microbiome under Stable Conditions but Are Critical for Recovery from Antibiotic Treatment | Experimental study | Mouse |
| 8 | Daytime Restricted Feeding Promotes Circadian Desynchrony and Metabolic Disruption with Changes in Bile Acids Profiles and Gut Microbiota in C57BL/6 Male Mice | Experimental study | Mouse |
| 9 | Abnormal Food Timing and Predisposition to Weight Gain: Role of Barrier Dysfunction and Microbiota | Experimental study | Mouse |
| 10 | The Intestinal Microbiota Programs Diurnal Rhythms in Host Metabolism through Histone Deacetylase 3 | Experimental study | Mouse |
| 11 | Short-Chain Fatty Acids Stimulate Colonic Transit via Intraluminal 5-HT Release in Rats | Experimental study | Rat |
| 12 | Indigenous Bacteria from the Gut Microbiota Regulate Host Serotonin Biosynthesis | Experimental study | Mouse |
| 13 | Late-Night Eating-Induced Physiological Dysregulation and Circadian Misalignment Are Accompanied by Microbial Dysbiosis | Experimental study | Mouse |
| 14 | Melatonin Orchestrates Lipid Homeostasis through the Hepatointestinal Circadian Clock and Microbiota during Constant Light Exposure | Experimental study | Mouse |
| 15 | The Circadian Disruption of Night Work Alters Gut Microbiota Consistent with Elevated Risk for Future Metabolic and Gastrointestinal Pathology | Observational study | Human |
| 16 | The Effects of Time-Restricted Eating on Metabolism and Gut Microbiota: A Real-Life Study | Interventional study | Human |
| 17 | The Effects of Artificial Light at Night on Eurasian Tree Sparrow (Passer Montanus): Behavioral Rhythm Disruption, Melatonin Suppression and Intestinal Microbiota Alterations | Experimental study | Bird (Sparrow) |
| 18 | Blue Light from Light-Emitting Diodes Elicits a Dose-Dependent Suppression of Melatonin in Humans | Experimental study | Human |
| 19 | Brief Disruption of Circadian Rhythms Alters Intestinal Barrier Integrity and Modulates DSS-Induced Colitis Severity in Mice | Experimental study | Mouse |
| 20 | The Pittsburgh Sleep Quality Index: A New Instrument for Psychiatric Practice and Research | Validation study | Human |
| 21 | Sleep, Activity, and Diet in Harmony: Unveiling the Relationships of Chronotype, Sleep Quality, Physical Activity, and Dietary Intake | Cross-sectional study | Human |
| 22 | Stress and Depression-Associated Shifts in Gut Microbiota: A Pilot Study of Human Pregnancy | Pilot study | Human |
| 23 | Effect of Time-Restricted Feeding on Metabolic Risk and Circadian Rhythm Associated with Gut Microbiome in Healthy Males | Randomized trial | Human |
| 24 | Circadian Rhythm Disruption Aggravates DSS-Induced Colitis in Mice with Fecal Calprotectin as a Marker of Colitis Severity | Experimental study | Mouse |
| 25 | Elevated Fecal Calprotectin in Patients with Alzheimer’s Dementia Indicates Leaky Gut | Observational study | Human |
| 26 | Cortisol Diurnal Rhythm and Stress Reactivity in Constipation and Abdominal Pain: The Generation R Study | Observational study | Human |
| 27 | Circadian Rhythm Modulation in Heart Rate Variability as Potential Biomarkers for Major Depressive Disorder: A Machine Learning Approach | Analytical study (machine learning) | Human |
| 28 | Nocturnal Heart Rate Variability Parameters as Potential Fibromyalgia Biomarker: Correlation with Symptoms Severity | Observational study | Human |
| 29 | [Autonomic nervous system activity in IBS patients estimated by heart rate variability (HRV)] | Observational study | Human |
| 30 | Competitive Enzyme Linked Aptamer Based Assay for Salivary Melatonin Detection | Assay development | In vitro |
| 31 | Melatonin Improves Skin Barrier Damage Caused by Sleep Restriction through Gut Microbiota | Experimental study | Mouse |
| 32 | Association between Circadian Rhythm Disruption and Polycystic Ovary Syndrome | Observational study | Human |
| 33 | Allicin Improves Diet-Induced Nonalcoholic Steatohepatitis and Gut Microbiota Dysbiosis in Mice via the Involvement of the Circadian Clock Gene Rev-Erbα | Experimental study | Mouse |
| 34 | Correlation of the Expression of Circadian-Clock Genes with the Severity of Obstructive Sleep Apnea in Patients | Observational study | Human |
| 35 | Associations between Plasma Proteomic Signatures and Secondary Sleep in Older Adults | Observational study | Human |
| 36 | Acute Sleep Loss Increases Circulating Morning Levels of Two MicroRNAs Implicated in Neurodegenerative Disease in Healthy Young Men | Experimental study | Human |
| 37 | Chronodisruption That Dampens Output of the Central Clock Abolishes Rhythms in Metabolome Profiles and Elevates Acylcarnitine Levels in the Liver of Female Rats | Experimental study | Rat |
| 38 | Twenty-Four-Hour Skin Temperature Rhythms in Young People With Emerging Mood Disorders: Relationships With Illness Subtypes and Clinical Stage | Observational study | Human |
| 39 | The Circadian Clock Regulates the Diurnal Levels of Microbial Short-Chain Fatty Acids and Their Rhythmic Effects on Colon Contractility in Mice | Experimental study | Mouse |
| 40 | Prebiotics Chronotherapy Alleviates Depression-like Behaviors in FMT Mice through Enhancing Short-Chain Fatty Acids Receptors and Intestinal Barrier | Experimental study | Mouse |
| 41 | Dietary Oat β-Glucan Alleviates High-Fat Induced Insulin Resistance through Regulating Circadian Clock and Gut Microbiome | Experimental study | Mouse |
| 42 | Chronic Restraint Stress Affects the Diurnal Rhythms of Gut Microbial Composition and Metabolism in a Mouse Model of Depression | Experimental study | Mouse |
| 43 | Shorter Sleep Time Relates to Lower Human Defensin 5 Secretion and Compositional Disturbance of the Intestinal Microbiota Accompanied by Decreased Short-Chain Fatty Acid Production | Cross-sectional study | Human |
| 44 | Effects of Prebiotics on Intestinal Physiology, Neuropsychological Function, and Exercise Capacity of Mice with Sleep Deprivation - PubMed | Experimental study | Mouse |
| 45 | Acute Sleep Deprivation Exacerbates Systemic Inflammation and Psychiatry Disorders through Gut Microbiota Dysbiosis and Disruption of Circadian Rhythms | Experimental study | Mouse |
| 46 | Identification of Preventive Biomarkers Associated with Circadian Rhythms in Traumatic Brain Injury-Mediated Depression: Expression of SERPINE1 Protein and Bioinformatics Analysis | Bioinformatics/analytical study | Mouse/Human |
| 47 | Development of a Circadian-Related Prognostic Signature Highlights RBM17 as a Stemness Regulator in Liver Cancer | Bioinformatics study | Human |
| 48 | Effects of 4-Hydroxy-2,3,3’,4’,5-Pentachlorobiphenyl (4-OH-CB107) on Liver Transcriptome in Rats: Implication in the Disruption of Circadian Rhythm and Fatty Acid Metabolism | Experimental study | Rat |
| 49 | Transgenerational Hepatotoxicity Induced by Bisphenol B as a Substitute for Bisphenol A | Experimental study | Mouse |
| 50 | Artificial Light at Night Accelerates Aging Processes in Pre-Pubertal Female Rats | Experimental study | Rat |
| 51 | Adrenal Hormone Response and Psychophysiological Correlates under Psychosocial Stress in Individuals with Irritable Bowel Syndrome | Observational study | Human |
| 52 | The Gut Microbiota-Derived Metabolite Trimethylamine N-Oxide Is Elevated in Alzheimer’s Disease | Observational study (cohort) | Human |
| 53 | Porphyromonas Gingivalis Induces Disturbance of Kynurenine Metabolism Through the Gut-Brain Axis: Implications for Alzheimer’s Disease | Experimental study | Mouse |
| 54 | Gut Bacterial Isoamylamine Promotes Age-Related Cognitive Dysfunction by Promoting Microglial Cell Death | Experimental study | Mouse |
| 55 | Effects of Long-Term Fasting on Gut Microbiota, Serum Metabolome, and Their Association in Male Adults | Experimental study | Human |
| 56 | Circadian Rhythm Perturbation Causes IBS-like Characteristics and Altered Fecal Metabolome in Mice | Experimental study | Mouse |
| 57 | Multi-Omics Assessment of Gut Microbiota in Circadian Rhythm Disorders: A Cross-Sectional Clinical Study | Cross-sectional study | Human |
| 58 | Fermented Gastrodia Elata Bl. Intervenes Gut Microbiota and Amino Acid Metabolism in Zebrafish to Promote 5-HT Homeostasis against Sleep Disturbances | Experimental study | Zebrafish |
| 59 | Molecular Mechanisms of Photoperiod Regulation of Bile Acid Metabolism in Taihe Silky Fowls Based on the Gut-Liver Axis | Experimental study | Chicken |
| 60 | The Microbiome Interacts with the Circadian Clock and Dietary Composition to Regulate Metabolite Cycling in the Drosophila Gut | Experimental study | Drosophila |
| 61 | Distinct Intestinal Microbial Signatures Linked to Accelerated Systemic and Intestinal Biological Aging | Observational study | Human |
| 62 | Gut Microbiota Regulates Stress Responsivity via the Circadian System | Experimental study | Mouse |
| 63 | Time-Restricted Feeding Affects Colonic Nutrient Substrates and Modulates the Diurnal Fluctuation of Microbiota in Pigs | Experimental study | Pig |
| 64 | Evening Use of Light-Emitting eReaders Negatively Affects Sleep, Circadian Timing, and next-Morning Alertness | Experimental study | Human |
| 65 | Blocking Nocturnal Blue Light for Insomnia: A Randomized Controlled Trial | Randomized controlled trial | Human |
| 66 | Association among Objective and Subjective Sleep Duration, Depressive Symptoms and All-Cause Mortality: The Pathways Study | Observational study (cohort) | Human |
| 67 | An Integrative Analysis of Cell-Specific Transcriptomics and Nuclear Proteomics of Sleep-Deprived Mouse Cerebral Cortex | Experimental study | Mouse |
| 68 | Longitudinal Analysis of the Immunostimulatory Properties and Safety Profile of Lacticaseibacillus Rhamnosus LRa05 as a Dietary Supplement | Experimental study | Mouse |
| 69 | Metagenomic Analysis Reveals the Signature of Gut Microbiota Associated with Human Chronotypes | Metagenomic/observational study | Human |
| 70 | Aging, Melatonin Biosynthesis, and Circadian Clockworks in the Gastrointestinal System of the Laboratory Mouse | Experimental study | Mouse |
| 71 | Resynchronized Rhythmic Oscillations of Gut Microbiota Drive Time-Restricted Feeding Induced Nonalcoholic Steatohepatitis Alleviation | Experimental study | Mouse |
| 72 | Randomized Controlled Trial for Time-Restricted Eating in Healthy Volunteers without Obesity | Randomized controlled trial | Human |
| 73 | Melatonin Alleviates Depression-like Behaviors and Cognitive Dysfunction in Mice by Regulating the Circadian Rhythm of AQP4 Polarization | Experimental study | Mouse |
| 74 | Regulation of Diel Locomotor Activity and Retinal Responses of Anopheles Stephensi by Ingested Histamine and Serotonin Is Temperature- and Infection-Dependent | Experimental study | Insect (Mosquito) |
